# Evaluation of the prevalence of MASLD, MASH and liver fibrosis in a Dutch bariatric cohort

**DOI:** 10.1371/journal.pone.0324813

**Published:** 2025-06-24

**Authors:** Willy Theel, Willem-Pieter Brouwer, Elisabeth van Rossum, Jan Apers, Susan ter Borg, Tessa Noordermeer, Manuel Castro Cabezas

**Affiliations:** 1 Department of Internal medicine, Franciscus Gasthuis & Vlietland, Rotterdam, the Netherlands; 2 Obesity Center CGG, Rotterdam, the Netherlands; 3 Department of Internal medicine, division of endocrinology, Erasmus MC, University Medical Center, Rotterdam, Rotterdam, the Netherlands; 4 Department of Gastroenterology & Hepatology, Erasmus MC, University Medical Center, Rotterdam, the Netherlands; 5 Department of bariatric surgery, Franciscus Gasthuis & Vlietland, Rotterdam, the Netherlands; 6 Department of Pathology, Franciscus Gasthuis & Vlietland, Rotterdam, the Netherlands; 7 Department of Clinical chemistry, Franciscus Gasthuis & Vlietland, Rotterdam, the Netherlands; 8 Julius Clinical, Zeist, the Netherlands; University of Hong Kong, Queen Mary Hospital, HONG KONG

## Abstract

**Background and aims:**

The prevalence of metabolic dysfunction-associated steatotic liver disease (MASLD) and metabolic dysfunction-associated steatohepatitis (MASH) in bariatric populations has been widely studied but may vary geographically. This study evaluates MASLD/MASH prevalence and the utility of non-invasive tests (NITs) for liver fibrosis in a Dutch bariatric surgery cohort.

**Methods:**

This single-center cross-sectional diagnostic accuracy study included 220 patients undergoing bariatric surgery. At baseline, 10 NITs were performed. Patients with liver stiffness measurements ≥ 8kPa using vibration-controlled transient elastography underwent a liver biopsy during surgery. Histology was assessed using the nonalcoholic fatty liver disease Activity Score. Diagnostic accuracy of NITs was evaluated against histology using sensitivity, specificity, and area under the receiver operating characteristic (AUROC). MASH was defined as steatosis with lobular inflammation and ballooning, with or without fibrosis. At-risk MASH included fibrosis ≥F2.

**Results:**

Out of 77 patients (35%) eligible for histological analysis, the findings revealed a MASLD prevalence of 50.6%, MASH prevalence of 5.3%, and at-risk MASH prevalence of 2.6%. Most patients had no fibrosis (67.1%), while others exhibited mild fibrosis (F1: 23.7%, F2: 9.2%). Capped MAF-5 effectively identified fibrosis stage ≥2 (AUROC: 0.809), surpassing FIB-4 (AUROC: 0.645). Both the FAST score and capped MAF-5 demonstrated strong performance in detecting at-risk MASH.

**Conclusion:**

MASLD/MASH prevalence and advanced fibrosis were lower than expected in this Dutch cohort. Capped MAF-5 demonstrated superior performance for fibrosis detection, while transient elastography and FIB-4 were less reliable. Further studies are needed to optimize NIT selection in bariatric populations.

## Introduction

Obesity is a worldwide epidemic with an increasing prevalence. Recent data predict a rise of individuals with obesity up to 2 billion in 2035 [1]. This rise is closely linked to the development of obesity-related comorbidities [2]. A partial list includes conditions such as type 2 diabetes mellitus (T2D), cardiovascular disease (CVD) and metabolic dysfunction-associated steatotic liver disease (MASLD). The latter is the most common chronic liver disease and is also associated with T2D and other cardiometabolic risk factors [3]. MASLD encompasses a spectrum of liver conditions, ranging from simple steatosis to metabolic dysfunction-associated steatohepatitis (MASH), which can progress to liver fibrosis and cirrhosis [4]. It is associated with hepatic events such as chronic liver failure and hepatocellular carcinoma (HCC) but also with extra hepatic outcomes like chronic kidney disease, cardiovascular disease, heart failure or colorectal carcinoma [5–9]. At least, 90% of the patients with severe obesity undergoing bariatric surgery may have MASLD [10,11] but data on European subjects are scarce [12,13].

The global burden of MASLD is high. High-risk groups, such as patients with obesity should undergo active screening for liver fibrosis associated with MASLD. Consequently, three scientific societies, the European Association for the Study of Liver, the European Association for the Study of Diabetes, and the European Association for the Study of Obesity, have proposed a strategy for non-invasive assessment of the risk for advanced fibrosis [14]. Although liver biopsies provide valuable information, they are not frequently performed due to associated risks and sampling error. Vibration controlled transient elastography (VCTE) is a good alternative for early diagnosis of liver fibrosis and has shown high accuracy in people with obesity [15]. The severity of liver fibrosis determines the risk of liver-related and cardiovascular outcomes [16].

At present, resmetirom is the only approved drug for treating adults with MASLD in the US. Despite the availability of medication, the primary treatment for MASLD remains weight loss and bariatric surgery is the most effective intervention for reducing body weight [17,18]. Multi-disciplinary experts agree that bariatric surgery should be recommended for individuals with MASH and severe obesity, while they also acknowledge that MASH or advanced liver fibrosis increase the risk of complications [19].

In this study, we aim to investigate the prevalence of MASLD and MASH in a Dutch cohort scheduled for bariatric surgery and assess the diagnostic value of noninvasive tests (NITs) to determine fibrosis risk in this population. These objectives will be addressed through the Franciscus Obesity NASH Study, as previously described [20].

## Methods and analysis

### Participants

Participants were recruited from the regular bariatric surgery program from the Obesity Center of the Franciscus Gasthuis Hospital in Rotterdam, the Netherlands, between 1 August 2022 and 15 November 2023. Participants fulfilled the criteria of the International Federation for the Surgery of Obesity and Metabolic Disorders at screening [21]. To participate in this study, subjects had to speak and read Dutch in order to provide written informed consent. This study was conducted according to the principles of the Declaration of Helsinki (version 2013), the Guideline for Good Clinical Practice (E6(R2)) and in accordance with the Medical Research Involving Human Subjects Act (WMO) and General Data Protection Regulation. The study was approved by the regional Medical Ethics Committee (MEC-U, Utrecht, The Netherlands) and the Institutional Review Board (IRB) of Stichting Sint Franciscus Vlietland Groep. The study is registered in the International Clinical Trial Registry Platform (ICTRP) (NL79423.100.21).

Patients who met any of the following criteria at screening could not take part to the study: (1) participants younger than 18 years or older than 65 years, (2) known with an established diagnosis of another primary liver disease, (3) alcohol consumption (>20g alcohol/day for woman and >30 g alcohol/day for men as defined in the local protocol), (4) histologically documented liver cirrhosis at screening or in a historical biopsy, (5) known with active HIV infection and/or treatment and (6) known with any type of diagnosed malignancies with or without active treatment.

### Bariatric surgery

The type of surgery—laparoscopic sleeve gastrectomy (LSG), Roux-en-Y gastric bypass (RYGB), one-anastomosis gastric bypass (OAGB), or Nissen sleeve gastrectomy (NSG)—was determined by the surgeon in consultation with the patient. There was no randomization of the procedure allocation.

### Outcome measures

Clinical data such as anthropometric measurements, blood tests and medical history were recorded.

The main outcome is to evaluate the prevalence of MASLD and the severity of liver fibrosis in a bariatric cohort. According to the available data, we evaluated the degree of liver fibrosis using TE, the Fibrosis-4 (FIB-4) index [22,23], the baseline metabolic dysfunction-associated fibrosis 5 (MAF-5) score [24], the capped MAF-5 score at BMI > 35 and over [25], aspartate aminotransferase (AST) to platelet ratio (APRI) [26], the nonalcoholic fatty liver disease (NAFLD) fibrosis score (NFS) [27], and the Fibroscan-based Agile scores [28]. These scores were defined as follows:


$$\begin{array}{c} \text{FIB - 4 index = (Age * AST)}/(\text{platelets}*\sqrt{}\text{ALT}) \end{array}$$

$$\begin{array}{c} \begin{array}{c} \text{Baseline MAF - 5 = - 11,3674 + waist circumference}*0,0282–\text{BMI x}0,1761\\ +\text{waist circumference}*\text{BMI x}0,0019+2,0762*\text{diabetes + ln(AST)}*2,9207–\text{platelets}*0,0059 \end{array} \end{array}$$

$$\begin{array}{c} \begin{array}{c} \text{Capped MAF - 5 = - 11,3674 + waist circumference}*0,0282–35\text{x}0,1761\\ +\text{waist circumference}*35\text{x}0,0019+2,0762*\text{diabetes + ln(AST)}*2,9207–\text{platelets}*0,0059 \end{array} \end{array}$$

$$\begin{array}{c} \text{APRI = (AST/(AST(ULN)) / platelets} \end{array}$$

$$\begin{array}{c} \text{NFS = - 1,675 + 0,037 * Age + 0,094 * BMI + 1,13 * diabetes}\\ +0,99*\text{AST/ALT–0,013*platelets–0,66*albumin} \end{array}$$

$$\begin{array}{c} \begin{array}{c} \text{Agile}3+=\hat{e}(-\mathrm{.3},92368+2,29714*\text{ln (LSM) – 0,00902 * platelets}–0,98633\\ {}*\text{AST/ALT + 1,08636 * diabetes – 0,38581 * male + 0,03018 * age})/\\ (1+\hat{e}(-\mathrm{.3},92368+2,29714*\text{ln (LSM) – 0,00902 * platelets}–0,98633\\ {}*\text{AST/ALT + 1,08636 * diabetes – 0,38581 * male + 0,03018 * age})) \end{array} \end{array}$$

$$\begin{array}{c} \begin{array}{c} \text{Agile}4=\hat{e}(7,50139–15,42498*1/\sqrt{}\text{LSM – 0,01378 * platelets}–1,41149\\ {}*\text{ALT/AST – 0,53281 * male + 0,41741 * diabetes)}/(1+\hat{e}(7,50139–15,42498\\ {}*1/\sqrt{}\text{LSM – 0,01378 * platelets – 1,41149 * ALT/AST – 0,53281 * male + 0,41741 * diabetes})) \end{array} \end{array}$$

The capped MAF-5 is identical to the baseline MAF-5, but it applies a BMI cap of 35 kg.m^-2^ to reduce false positive cases resulting from overestimation caused by a high BMI. An elevated FIB-4 value of ≥1.3, a baseline and capped MAF-5 value of ≥0, an APRI ≥0,918 or a NFS > 0,676 were used as a surrogate marker for significant liver fibrosis (F2) as defined by Kleiner et al. [29]. A cutoff value of 0,679 was applied to rule in advanced fibrosis (F3) using the Agile 3 + score, while a cutoff value of 0,565 was used to rule in cirrhosis (F4) with the Agile 4 score, as proposed by Sanyal et al. [28].

### Fibroscan measurements and MASLD definition

VCTE (Fibroscan^®^, Echosens SA, Paris, France) with liver-controlled attenuation parameter (CAP) was used to quantify liver steatosis, while liver stiffness measurement (LSM) was used to assess the degree of liver fibrosis. A CAP ≥ 275 dB/m was chosen to define MASLD and a LSM ≥ 8 kPa defined significant fibrosis.

According to manufacturer’s instructions, VCTE was performed on the lying participants, placing the probe in the intercostal space, close to the right lobe of the liver [30,31]. At least ten valid measurements were done by an operator trained and certified by Echosens. As published elsewhere, the interquartile range – median ratio had to be < 30% to consider the measurements as successful [32]. Outside these conditions, the measurements were deemed failures and were not used for analysis. VCTE was performed no more than two weeks prior to surgery.

According to the literature available at the time of protocol submission, a preoperative liver elasticity ≥ 7.95 kPa was chosen as the threshold for performing a perioperative liver biopsy. The biopsy was obtained from the diaphragmatic surface of segment three or five of the liver and the size varied between 1 and 3 cm in length and between 1,2 and 2 mm in diameter. Biopsies were assessed for histology (paraffin embedded) and two pathologists determined and individually scored the MASLD/MASH status using the NAFLD Activity Score (NAS) [29]. In case of disagreement on the classification, a third pathologist was consulted and the score set by 2 out of 3 pathologists was recorded.

Participants with liver steatosis >5% automatically met the definition of MASLD due to the presence of obesity as a cardiometabolic risk factor. Both a clinical approach using LSM and a histological approach with liver biopsies were employed to analyze MASLD in our cohort.

MASH was histologically defined by the presence of steatosis, lobular inflammation, and ballooning, with a NAS score of ≥4, with or without fibrosis. Cases with significant or advanced fibrosis (F ≥ 2) on histological evaluation were classified as at-risk MASH.

At-risk MASH was predicted with a Fibroscan AST (FAST) score ≥0,35, as described by Newsome et al. [33], or a Fibrotic NASH index (FNI) of ≥0,33 [34].Therefore we used the following formulas:


$$\begin{array}{c} \begin{array}{c} \text{FAST}=\hat{e}(-1,65+1,07*\text{ln(LSM)}+2,66*10-8*\text{CAP3 – 63,3 * AST}-1)/\\ \left(1+\hat{e}(-1,65+1,07*\text{ln(LSM)}+2,66*10-8*\text{CAP3 – 63,3 * AST}-1)\right) \end{array} \end{array}$$

$$\begin{array}{c} \begin{array}{c} \text{FNI}=\hat{e}(-10,33+2,54*\text{ln(AST) + 3,86 * ln(HbA1c) – 1,66 * (HDL))}/\\ (1+\hat{e}(-10,33+2,54*\text{ln(AST) + 3,86 * ln(HbA1c) – 1,66 * (HDL)))} \end{array} \end{array}$$

At last, baseline and capped MAF-5 scores were also used in the evaluation of the detection of at-risk MASH cases.

### Sample size

A sample size calculation was performed based on previous studies including TE measurements, epidemiological data, and dropout rates during bariatric follow-up. The sample size calculation for the entire study has been previously conducted and extensively detailed in a prior publication [20].

### Statistical analysis

Continuous variables were evaluated using the two-tailed independent t-test when normally distributed and were expressed as means with standard deviations. Categorical variables were assessed using the Chi-square test and expressed in terms of frequency and percentage. Outliers were not removed. The diagnostic value of NITs was evaluated by calculating sensitivity, specificity, positive predictive value, negative predictive value, and the area under the receiver operating characteristic curve (AUROC), compared to the NAS score from biopsies. We used the highest Youden’s index to determine the best cut-off value from the AUROC. Statistical analysis was performed using IBM SPSS version 29 (IBM Corporation, Armonk, New York, USA). P values < 0,05 (two-tailed) were considered statistically significant.

## Results

### Baseline characteristics

A total of 220 patients were recruited between August 2022 and November 2023, all of whom underwent bariatric surgery with a valid preoperative VCTE assessment. The mean age was 43,2 years (±11,5), and the mean BMI was 42,9 kg/m² (±5,7). The majority were women (80,5%). Most patients were of Caucasian ethnicity (74,5%). At baseline, 36 patients (16,3%) had diabetes mellitus (1,8% with type 1 and 14,5% with type 2), 64 (29,1%) had hypertension, and 82 (37,3%) had dyslipidemia (see Table 1).

**Table 1:** Baseline characteristics of the FONS population based on VCTE and liver histology.

	Based on VCTE				Based on histology			
Demographics	Total	No MASLD	MASLD	P-value	Total	No MASLD	MASLD	P-value
n (%)	220 (100%)	105 (47,7%)	115 (52,3%)		77 (100%)	38 (49,3%)	39 (50,6%)	
Age, years ±SD	43,2 ± 11,5	40,7 ± 11,1	45,5 ± 11,4	0,002	44,7 ± 11,0	43,4 ± 12,2	45,7 ± 9,7	0,427
Women, n(%)	177 (80,5%)	92 (87,6%)	85 (73,9%)	0,010	58 (75,3%)	33 (86,8%)	25 (64,1%)	0,021
Height, cm	169,3 ± 8,2	168,5 ± 7,6	170,1 ± 8,6	0,158	170,3 ± 8,8	168,4 ± 9,3	172,3 ± 8,0	0,054
Weight, kg	123,3 ± 20,9	121,5 ± 19,0	124,8 ± 22,5	0,238	125,8 ± 21,5	122,0 ± 17,4	129,5 ± 24,5	0,127
Waist circumference, cm	130,5 ± 13,5	127,9 ± 13,4	132,7 ± 13,3	0,008	132,5 ± 13,2	131,7 ± 12,2	133,4 ± 14,1	0,565
BMI, kg.m^-2^	42,9 ± 5,7	42,7 ± 5,3	43,0 ± 6,00	0,674	43,2 ± 5,9	43,0 ± 4,5	43,5 ± 7,1	0,675
Etnicity, n (%)				0,486				0,212
Hindu	8 (3,6%)	5 (4,8%)	3 (2,6%)		2 (2,6%)	1 (2,6%)	1 (2,6%)	
Asian	1 (0,5%)	1 (1,0%)	0 (0%)		1 (1,3%)	1 (2,6%)	0 (0%)	
Caucasian	164 (74,5%)	73 (69,5%)	91 (79,1%)		57 (74,0%)	27 (71,1%)	30 (76,9%)	
Latino-american	2 (0,9%)	0 (0%)	2 (1,7%)		0 (0%)	0 (0%)	0 (0%)	
North African	9 (4,1%)	5 (4,8%)	4 (3,5%)		0 (0%)	0 (0%)	0 (0%)	
Other African	14 (6,4%)	9 (8,6%)	5 (4,3%)		9 (11,7%)	7 (18,4%)	2 (5,1%)	
Turkish	11 (5%)	6 (5,7%)	5 (4,3%)		3 (3,9%)	0 (0%)	3 (7,7%)	
Other	11 (5%)	6 (5,7%)	5 (4,3%)		5 (6,5%)	2 (5,3%)	3 (7,7%)	
Diabetes, n(%)	36 (16,3%)	15 (14,3%)	21 (18,3%)	0,426	21 (27,3%)	8 (21,1%)	13 (33,3%)	0,215
No diabetes	184 (83,6%)	90 (85,7%)	94 (81,7%)		56 (72,7%)	30 (78,9%)	26 (66,7%)	
Type 1	4 (1,8%)	3 (20,0%)	1 (4,8%)		1 (1,3%)	1 (2,6%)	0 (0%)	
Type 2	32 (14,5%)	12 (80,0%)	20 (95,2%)		20 (26,0%)	7 (18,4%)	13 (33,3%)	
Hypertension, n(%)	64 (29,1%)	25 (23,8%)	39 (33,9%)	0,099	30 (39,0%)	13 (34,2%)	17 (43,6%)	0,399
Dyslipidemia, n(%)	82 (37,3%)	40 (38,1%)	42 (36,5%)	0,809	30 (39,0%)	10 (26,3%)	20 (51,3%)	0,025
OSAS, n(%)	25 (11,4%)	14 (13,3%)	11 (9,6%)	0,379	5 (6,5%)	1 (2,6%)	4 (10,3%)	0,175
**Laboratory measurements**								
Creatinine, µmol/L	71,4 ± 28,8	68,8 ± 12,7	73,8 ± 37,8	0,197	75,9 ± 45,1	67,8 ± 8,7	83,9 ± 62,2	0,119
ALT, U/L	32,2 ± 21,5	29,0 ± 20,9	35,2 ± 21,6	0,032	35,8 ± 28,4	24,2 ± 9,4	47,0 ± 35,6	<0,001
AST, U/L	27,4 ± 11,6	26,1 ± 12,2	28,5 ± 11,0	0,133	30,0 ± 16,2	23,4 ± 4,4	36,2 ± 20,4	<0,001
Cholesterol, mmol/L	5,19 ± 1,03	5,06 ± 0,96	5,31 ± 1,09	0,068	5,1 ± 1,2	4,9 ± 1,1	5,3 ± 1,2	0,105
LDL-chol, mmol/L	3,18 ± 0,87	3,13 ± 0,83	3,23 ± 0,91	0,435	3,10 ± 0,92	3,01 ± 0,88	3,21 ± 0,97	0,349
HDL-chol, mmol/L	1,21 ± 0,30	1,24 ± 0,33	1,19 ± 0,26	0,217	1,16 ± 0,26	1,22 ± 0,27	1,10 ± 0,24	0,046
Chol/HDL	4,48 ± 1,25	4,32 ± 1,31	4,63 ± 1,17	0,069	4,50 ± 1,16	4,06 ± 0,87	4,92 ± 1,25	<0,001
Triglycerides, mmol/L	1,84 ± 1,15	1,57 ± 1,01	2,09 ± 1,21	<0,001	1,84 ± 1,14	1,40 ± 0,64	2,27 ± 1,35	<0,001
Transferrine, g/L	2,81 ± 0,39	2,75 ± 0,40	2,87 ± 0,39	0,022	2,83 ± 0,40	2,84 ± 0,47	2,81 ± 0,32	0,771
Ferritine, µg/L	110,3 ± 103,5	100,9 ± 106,1	119,0 ± 100,8	0,194	126,7 ± 111,9	89,2 ± 70,3	163,1 ± 132,2	0,003
Iron, umol/L	15,1 ± 5,6	14,8 ± 5,6	15,4 ± 5,6	0,381	14,9 ± 4,7	13,8 ± 4,4	16,0 ± 4,8	0,041
HbA1c, mmol/mol	41,5 ± 10,5	39,4 ± 9,4	43,4 ± 11,1	0,005	45,1 ± 13,9	42,6 ± 11,2	47,4 ± 15,8	0,132
Albumin, g/L	45,6 ± 2,6	45,4 ± 2,7	45,8 ± 2,5	0,260	45,9 ± 2,5	45,3 ± 2,5	46,5 ± 2,3	0,040
Hemoglobin, mmol/L	8,7 ± 0,9	8,6 ± 0,9	8,8 ± 0,8	0,030	8,8 ± 0,8	8,6 ± 0,8	8,9 ± 0,9	0,115
Hematocrite, L/L	0,04	0,42 ± 0,04	0,43 ± 0,04	0,013	0,43 ± 0,04	0,42 ± 0,04	0,44 ± 0,04	0,119
Platelets, x10^9^/L	301,2 ± 66,9	302,9 ± 61,4	299,6 ± 71,8	0,723	296,2 ± 74,5	299,1 ± 74,6	293,5 ± 75,3	0,742
White blood cells, x10^9^/L	8,2 ± 2,2	8,4 ± 2,3	8,1 ± 2,1	0,366	8,3 ± 2,2	8,7 ± 2,1	7,9 ± 2,3	0,144

Data are given as mean (SD).OSAS: obstructive sleep apnea syndrome; ALT: alanine transaminase; AST: aspartate transaminase; LDL: low density lipoprotein; HDL: high-density lipoprotein. Results are presented as numbers and percentages, or mean and standard deviation. When based on VCTE, MASLD was established based on a CAP value ≥275 dB/m.

The mean CAP was 283 dB/m (±50,5) and the mean LSM was 8.25 kPa (±4,49). A total of 42,3% of participants had an LSM ≥ 8 kPa. Table 2 shows the mean NIT scores.

**Table 2:** Results of noninvasive tests of the FONS cohort based on VCTE and liver histology.

	Based on VCTE				Based on histology			
Non invasive tests	Total	No MASLD	MASLD	P-value	Total	No MASLD	MASLD	P-value
N (%)	220 (100%)	105 (47,7%)	115 (52,3%)		77 (100%)	38 (49,3%)	39 (50,6%)	
FIB-4 index ± SD (=211)	0,768 ± 0,337	0,723 ± 0,344	0,801 ± 0,326	0,063	0,854 ± 0,396	0,812 ± 0,432	0,894 ± 0,361	0,382
< 1,3, n (%)	193 (91,5%)	94 (92,2%)	99 (90,8%)	0,197	63 (86,3%)	30 (85,7%)	33 (86,8%)	0,889
≥ 1,3 and <2,67, n (%)	18 (8,5%)	8 (7,8%)	10 (9,2%)		10 (13,7%)	5 (14,3%)	5 (13,2%)	
≥ 2,67, n (%)	0 (0%)	0 (0%)	0 (0%)		0 (0%)	0 (0%)	0 (0%)	
Baseline MAF-5 ± SD (=211)	3,284 ± 2,365	2,758 ± 2,440	3,777 ± 2,191	0,002	3,990 ± 2,505	3,125 ± 2,348	4,787 ± 2,405	0,004
< 0, n (%)	10 (4,7%)	9 (8,8%)	1 (0,9%)	<0,001	1 (1,4%)	1 (2,9%)	0 (0%)	0,172
≥ 0 and <1, n (%)	27 (12,8%)	19 (18,6%)	8 (7,3%)		10 (13,9%)	7 (20,0%)	3 (7,9%)	
≥ 1, n (%)	174 (82,5%)	74 (72,5%)	100 (91,7%)		61 (84,7%)	27 (77,1%)	35 (92,1%)	
Capped MAF-5 ± SD (=211)	−0,546 ± 1,742	−0,919 ± 1,811	−0,197 ± 1,605	0,002	0,042 ± 1,988	−0,686 ± 1,845	0,712 ± 1,897	0,002
< 0, n (%)	135 (64,0%)	72 (70,6%)	63 (57,8%)	0,153	38 (53,4%)	24 (68,6%)	15 (39,5%)	0,031
≥ 0 and <1, n (%)	36 (17,1%)	14 (13,7%)	22 (20,2%)		9 (12,3%)	4 (11,4%)	5 (13,2%)	
≥ 1, n (%)	40 (18,9%)	16 (15,7%)	24 (22,0%)		25 (34,2%)	7 (20,0%)	18 (47,4%)	
APRI ± SD (n = 211)	0,311 ± 0,205	0,285 ± 0,152	0,323 ± 0,191	0,116	0,349 ± 0,242	0,263 ± 0,093	0,428 ± 0,304	0,003
≥ 0,918, n (%)	5 (2,4%)	2 (2,0%)	3 (2,8%)	0,706	4 (5,5%)	0 (0%)	4 (10,5%)	0,048
NFS ± SD (n = 211)	−1,884 ± 1,238	−1,977 ± 1,262	−1,801 ± 1,233	0,307	−1,624 ± 1,328	−1,672 ± 1,488	−1,580 ± 1,180	0,769
> 0,676, n(%)	2 (0,9%)	1 (1,0%)	1 (0,9%)	0,962	1 (1,4%)	1 (2,9%)	0 (0%)	0,287
CAP, dB/m (=220)	283,00 ± 50,5	240,1 ± 27,1	322,2 ± 31,5	<0,001	291,7 ± 54,0	272,97 ± 49,5	310,0 ± 52,5	0,002
LSM, kPa ± SD (=220)	8,25 ± 4,49	7,74 ± 4,58	8,72 ± 4,37	0,109	11,77 ± 4,94	11,70 ± 4,87	11,84 ± 5,06	0,908
≥ 8 kPa, n(%)	93 (42,3%)	38 (36,2%)	55 (47,8%)	0,081	42 (56,8%)	19 (50,0%)	23 (63,9%)	0,320
IQR/median preoperative (%)	18,7 ± 6,6	18,6 ± 6,5	18,9 ± 6,6	0,692	18,9 ± 6,2	19,5 ± 5,7	18,4 ± 6,7	0,468
FAST score ± SD (n = 212)	0,24 ± 0,17	0,16 ± 0,13	0,31 ± 0,18	<0,001	0,34 ± 0,20	0,25 ± 0,13	0,43 ± 0,22	<0,001
≥ 0,35, n(%)	45 (21,2%)	10 (9,7%)	35 (32,1%)	<0,001	31 (41,9%)	7 (19,4%)	24 (63,2%)	<0,001
FNI ± SD	0,23 ± 0,22	0,18 ± 0,19	0,25 ± 0,19	0,013	0,29 ± 0,25	0,18 ± 0,17	0,39 ± 0,28	<0,001
≥ 0,33, n(%)	39 (18,6%)	13 (12,9%)	26 (23,9%)	0,041	21 (28,8%)	5 (14,3%)	16 (42,1%)	0,009
Agile3+ ± SD (n = 211)	0,237 ± 0,219	0,188 ± 0,192	0,283 ± 0,233	0,001	0,415 ± 0,229	0,382 ± 0,223	0,447 ± 0,232	0,226
≥ 0,679, n(%)	13 (6,2%)	3 (2,9%)	10 (9,2%)	0,060	11 (15,1%)	3 (8,6%)	8 (21,1%)	0,136
Agile 4 ± SD (n = 211)	0,041 ± 0,066	0,036 ± 0,064	0,046 ± 0,068	0,310	0,087 ± 0,093	0,100 ± 0,109	0,074 ± 0,074	0,224
≥ 0,565, n(%)	0 (0,0%)	0 (0,0%)	0 (0,0%)	NA	0 (0,0%)	0 (0,0%)	0 (0,0%)	NA

Data are given as mean (SD). APRI: AST to platelet ratio; CAP: controlled attenuation parameters; FAST score: Fibroscan-AST score; FNI: fibrotic NASH index; IQR: interquartile range; LSM: liver stiffness measurement; MAF-5: metabolic dysfunction-associated fibrosis 5; NFS: NAFLD fibrosis score; SD: standard deviation;. Results are presented as numbers and percentages, or mean and standard deviation. When based on VCTE, MASLD was established based on a CAP value ≥275 dB/m.

### Risk factors for MASLD

Based on VCTE, individuals with MASLD were significantly older, with the majority being male, and exhibited greater waist circumference despite having a comparable BMI, indicating central adiposity. They also had higher levels of ALT, triglycerides, and HbA1c, despite a comparable proportion of individuals with diabetes or dyslipidemia.

Based on histology, individuals with MASLD were predominantly male, but no differences were found in other demographic characteristics. The laboratory measurements were characterized by higher levels of transaminases, cholesterol/HDL ratio, triglycerides, ferritin, and iron, suggesting possible inflammation.

Baseline differences are shown in Table 1.

### Noninvasive liver tests based on MASLD status

In both the VCTE and histology groups, several parameters (baseline MAF-5, Capped MAF-5, CAP, FAST score, and FNI) demonstrated significant differences between individuals with and without MASLD. The APRI was higher in the histology-based MASLD group, with a greater percentage of patients with fibrosis stage F ≥ 2. The Agile 3 + score was also elevated in the TE-based MASLD group; however, the proportion of participants with F ≥ 3 did not differ significantly. Other parameters, such as FIB-4, NFS, LSM, and Agile 4, did not show significant differences between the groups. Results of the non-invasive tests (NITs) are presented in Table 2.

### Liver biopsy

A total of 77 liver biopsies were of sufficient quality for evaluation. Based on the histological analysis, 39 (50,6%) of them exhibit steatosis and therefore met the definition of MASLD. Only 4 of them (5,3%) met the definition of MASH of whom 2 (2,6%) were at-risk MASH. No fibrosis (F0) was found in 51 biopsies (67,1%), perisinusoidal or portal fibrosis (F1) in 18 biopsies (23,7%), and perisinusoidal and periportal fibrosis (F2) in 7 biopsies (9,2%). Neither septal or bridging fibrosis (F3), nor cirrhosis (F4), was found. Based on the TE distinction, only the proportion of individuals with steatosis was significantly higher in the MASLD group. However, 27 individuals (35,1%) were misclassified: 11 biopsies from the no-MASLD group exhibited steatosis, while 16 biopsies from the MASLD group did not. When using the histology for the MASLD classification, the MASLD group exhibited a higher proportion of participants with a NAS score ≥4 and MASH. These findings are shown in Table 3.

**Table 3:** Bariatric surgery and liver biopsies.

	Based on histology			
Surgery and liver biopsies	Total	No MASLD	MASLD	P-value
N (%)	77 (100%)	38 (49,3%)	39 (50,6%)	
Type of surgery				0,408
LSG	39 (50,6%)	21 (55,3%)	18 (46,2%)	
RYGB	36 (46,8%)	16 (42,1%)	20 (51,3%)	
OAGB	1 (1,3%)	1 (2,6%)	0 (0%)	
Nissen sleeve	1 (1,3%)	0 (0%)	1 (2,6%)	
NAS score ≥ 4, n(%)	6 (7,9%)	0 (0%)	6 (15,4%)	<0,001
MASH	4 (5,3%)	0 (0%)	4 (10,3%)	0,045
At-risk MASH	2 (2,6%)	0 (0%)	2 (5,1%)	0,163
Steatosis ≥ 1, n(%)	39 (50,6%)	0 (0%)	39 (100%)	
Balooning ≥ 1, n(%)	12 (15,6%)	3 (7,9%)	9 (23,1%)	0,074
Inflammation ≥ 1, n(%)	42 (54,5%)	19 (50,0%)	23 (59,0%)	0,504
Fibrosis				0,237
0, n(%)	51 (67,1%)	29 (76,3%)	22 (57,9%)	
1, n(%)	18 (23,7%)	7 (18,4%)	11 (28,9%)	
2, n(%)	7 (9,2%)	2 (5,3%)	5 (13,2%)	
3, n(%)	0 (0%)	0 (0%)	0 (0%)	
4, n(%)	0 (0%)	0 (0%)	0 (0%)	

LSG: laparoscopic sleeve gastrectomy; RYGB: Roux-en-Y gastric bypass; OAGB: one-anastomosis gastric bypass; NSG: Nissen sleeve gastrectomy. Results are presented as numbers and percentages. When based on TE, MASLD was established based on a CAP value ≥275 dB/m.

### Evaluation of non-invasive tests

In order to evaluate the diagnostic value of the NITs, we compared them with the set of liver biopsies.

The difference in mean values of NITs between F0-1 and ≥F2 was significant different for, the baseline and capped MAF-5 scores and NFS but not for the FIB-4, APRI or TE. However, only the capped MAF-5 and NFS made a significant distinction between these fibrosis stages. The capped MAF-5 showed a sensitivity of 100% while the NFS a specificity of 100%. In practice, the capped MAF-5 score could be used to screen and rule out F2 and NFS could confirm the diagnosis in positive cases.

Due to the absence of F3 and F4 cases in our biopsy set, we were unable to calculate the test characteristics for Agile 3+ and Agile 4. The Agile 3 + still identified 10 individuals as possibly having F3 fibrosis.

Finally, both MAF-5 scores, the FAST score and FNI showed different mean values between individuals with and without at-risk MASH. These tests are useful for ruling out at-risk MASH; however, further investigation is required in cases of positive results. The tests characteristics are presented in Table 4.

**Table 4:** Evaluation of the NITs: tests characteristics.

NIT’s	F0-1	≥F2	p-value	Sensitivity	Specificity	PPV	NPV
FIB-4 index	0,827 ± 0,364	1,141 ± 0,596	0,217	28,6%	87,7%	0.2	0,919
< 1.3, n (%)	57 (87,7%)	5 (71,4%)					
≥ 1.3, n (%)	8 (12,3%)	2 (28,6%)	0,237				
Baseline MAF-5	3,681 ± 2,390	6,423 ± 2,203	0,005	100%	1,5%	0,099	1
< 0, n (%)	1 (1,5%)	0 (0,0%)					
≥ 0, n(%)	64 (98,5%)	7 (100,0%)	0,741				
Capped MAF-5	−0,192 ± 1,958	1,808 ± 0,994	0,010	100%	60%	0,212	1
< 0, n (%)	39 (60,0%)	0 (0,0%)					
≥ 0, n(%)	23 (40,0%)	7 (100,0%)	0,002				
APRI ± SD	0,325 ± 0,212	0,578 ± 0,391	0,140	28,6%	96,9%	0,5	0,926
< 0,918, n (%)	63 (96,9%)	5 (71,4%)					
≥ 0,918, n (%)	2 (3,1%)	2 (28,6%)	0,005				
NFS ± SD	−1,724 ± 1,324	−0.594 ± 1,021	0,032	14,3%	100%	1	0,915
≤ 0,676, n(%)	65 (100,0%)	6 (85,7%)					
> 0,676, n(%)	0 (0,0%)	1 (14,3%)	0.002				
LSM	11,45 ± 4,27	13,51 ± 8,95	0,567	100%	1,5%	0,093	1
< 8 kPa, n(%)	1 (1,4%)	0 (0,0%)					
≥ 8 kPa, n(%)	68 (98,6%)	7 (100,0%)	0,748				
**NIT’s**	**<F3**	**≥F3**	**p-value**	**Sensitivity**	**Specificity**	**PPV**	**NPV**
Agile 3+	0.411 ± 0.228	NA	NA	NA	NA	NA	NA
< 0,679, n(%)	62 (86,1%)	0 (0,0%)					
≥ 0,679, n(%)	10 (13,9%)	0 (0,0%)	NA				
**NIT’s**	**<F4**	**F4**	**p-value**	**Sensitivity**	**Specificity**	**PPV**	**NPV**
Agile 4	0,0874 ± 0,093	NA	NA	NA	NA	NA	NA
< 0,565, n(%)	72 (100,0%)	0 (0,0%)					
≥ 0,565, n(%)	0 (0.0%)	0 (0,0%)	NA				
**NIT’s**	**Not At-risk MASH**	**At-risk MASH**	**p-value**	**Sensitivity**	**Specificity**	**PPV**	**NPV**
Baseline MAF-5	3,929 ± 2,507	6,785 ± 0,864	0,114	100%	1,4%	0,028	1
< 0, n (%)	1 (1,4%)	0 (0,0%)					
≥ 0, n(%)	69 (98,6%)	2 (100,0%)	0,865				
Capped MAF-5	−0,024 ± 1,966	2,884 ± 0,256	0,041	100%	54,2%	0,06	1
< 0, n (%)	38 (54,3%)	0 (0,0%)					
≥ 0, n(%)	32 (45,7%)	2 (100,0%)	0,129				
FAST score	0,33 ± 0,19	0,73 ± 0,13	0,005	100%	60,6%	0,067	1
< 0,35, n(%)	43 (60,6%)	0 (0,0%)					
≥ 0,35, n(%)	28 (39,4%)	2 (100,0%)	0,086				
FNI	0,28 ± 0,24	0.76 ± 0,17	0,003	100%	72,9%	0,095	1
< 0,33, n(%)	51 (72,9%)	0 (0,0%)					
≥ 0,33, n(%)	19 (27,1%)	2 (100,0%)	0,025				

APRI: AST to platelet ratio; CAP: controlled attenuation parameters; FAST score: Fibroscan-AST score; FNI: fibrotic NASH index; IQR: interquartile range; LSM: liver stiffness measurement; MAF-5: metabolic dysfunction-associated fibrosis 5; NFS: NAFLD fibrosis score; NPV: negative predictive value; PPV: positive predictive value; SD: standard deviation;. Results are presented as numbers and percentages, or mean and standard deviation.

**Fig 1:**
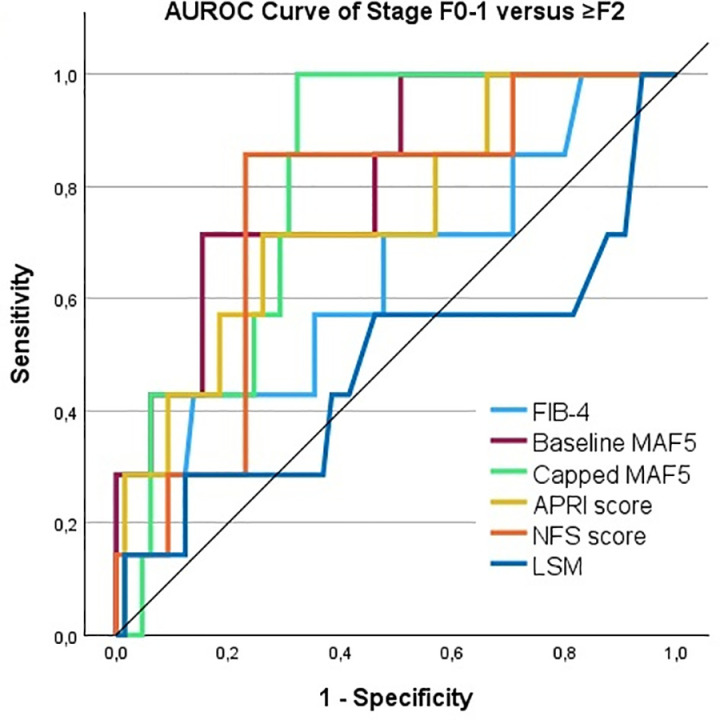
AUROC curves of NIT’s. AUROC curves from (A) FIB-4 index, baseline MAF-5, capped MAF-5, APRI score, NFS score and Fibroscan®(LSM) for the detection of stage F ≥ 2 and. The performance of these tests was evaluated against histology. The diagonal line corresponds to chance alone (AUROC curve = 0.50).

**Fig 2:**
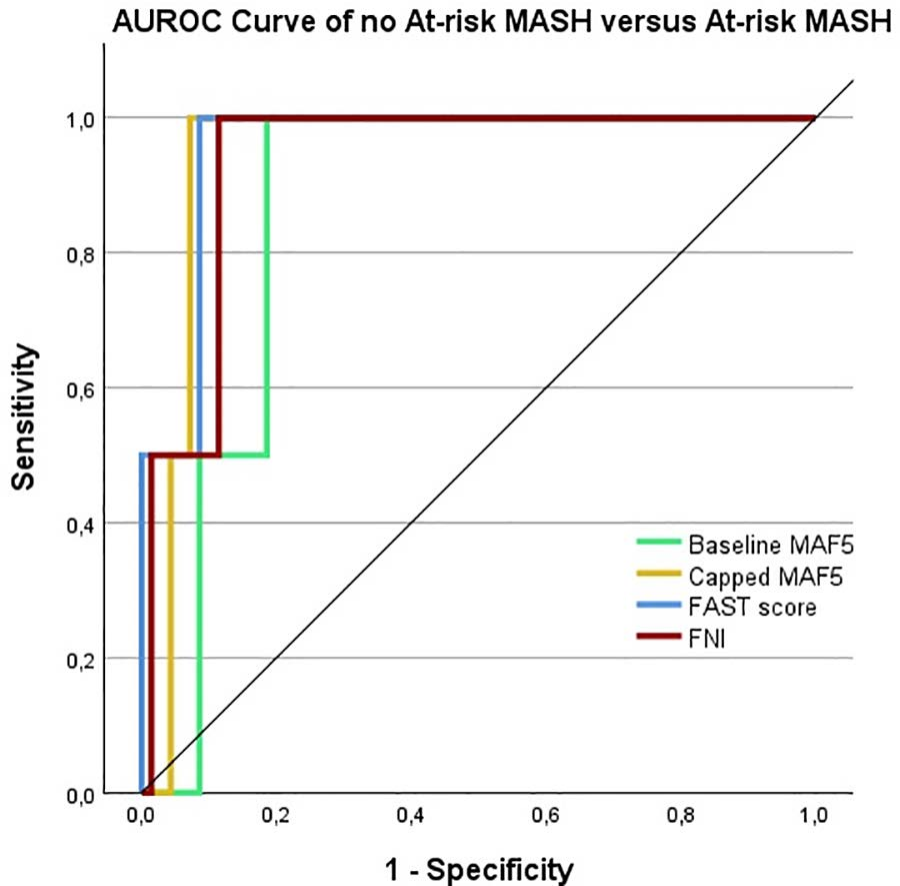
AUROC curves of NIT’s. AUROC curves from Baseline MAF-5, Capped MAF-5, FAST score and FNI for the detection of at-risk MASH. The performance of these tests was evaluated against histology. The diagonal line corresponds to chance alone (AUROC curve = 0.50).

The AUROCs of the tests (excluding the Agile tests) are shown in Table 5 and visualized in Fig 1–2. Both baseline and capped MAF-5 share the highest AUROC of 0.809, making them the top performers for detecting F ≥ 2. The best two test for detecting at-risk MASH were the FAST score and the capped MAF-5 scores, demonstrating AUROCs of 0.957 and 0.943, respectively. The optimal cutoff values for each test were defined using the highest Youden’s index.

**Table 5:** AUROCs and optimal cutoff values for each test in detecting F ≥ 2 and At-risk MASH.

Aera Under the ROC Curve of Stage F0-1 versus ≥F2			
FIB-4 index	0,645		
Baseline MAF-5	0,809		
Capped MAF-5	0,809		
APRI score	0,743		
NFS score	0,754		
LSM	0,479		
Aera Under the ROC Curve of no At-risk MASH versus At-risk MASH			
Baseline MAF-5	0,864		
Capped MAF-5	0,943		
FAST score	0,957		
FNI	0,936		
Optimal cutoff values for ≥F2	cutoff value	sensitivity	specificity
FIB-4 index	1,26	42,90%	13,80%
Baseline MAF-5	6,043	71,40%	15,40%
Capped MAF-5	0,475	100%	32,30%
APRI score	0,351	71,40%	26,20%
NFS score	−0,708	85,70%	23,10%
LSM	14,3	28,60%	12,30%
Optimal cutoff values for At-risk MASH	cutoff value	sensitivity	specificity
Baseline MAF-5	6,15	100%	18,60%
Capped MAF-5	2,69	100%	7,10%
FAST score	0,63	100%	8,60%
FNI	0,62	100%	11,40%

## Discussion

In this study investigating the prevalence of MASLD and MASH in a Dutch bariatric cohort, 52.3% of patients had MASLD based on VCTE. Based on histology in a subset of the patients, 50.6% had MASLD, while 5.3% had MASH. Among the patients who underwent biopsy, the majority (67.1%) had no fibrosis, while a smaller percentage exhibited mild fibrosis (F1: 23.7%, F2: 9.2%). No patients were diagnosed with F3 fibrosis or cirrhosis. Additionally, 2.6% of patients had at-risk MASH.

The capped MAF-5 and NFS successfully ruled out F2 and confirmed the diagnosis, where other NITs failed to reliably determine liver fibrosis. In our study, the capped MAF-5 was the most effective NIT for predicting fibrosis stage ≥2, with an AUROC of 0.809. The FAST score and capped MAF-5 showed a great AUROC of 0.957 and 0.943 respectively.

These findings differ from previous studies. Recent results from similar populations have been published in various countries, including Germany, China, France, Italy, and Brazil [12,13,35–37]. Although the preoperative mean LSM of 8.25 kPa in our study is consistent with previous reports ranging from 7.01 to 12.9 kPa, there is a substantial difference in the distribution of fibrosis categories. In a 2023 multicenter, open-label, randomized trial in Italy including 431 liver biopsies, 98.7% of patients had liver fibrosis ≥F1, with 42.4% having ≥F2 [37]. Higher LSM values were also found by others including 101 patients in China, where 64.9% of patients had liver fibrosis [13]. Similarly, a French group reported 62% of patients with liver fibrosis in their cohort including 100 liver biopsies [36]. In our study no patients were diagnosed with F3 fibrosis or cirrhosis, in contrast to a German study where 48% of the patients had bridging fibrosis or cirrhosis prior to bariatric surgery [35]. Finally, our results align more closely with those of Raverdy et al. [38], who reported that 72% of patients had no liver fibrosis prior to bariatric surgery in a French cohort of 2.436 individuals. While they diagnosed patients with F3 and F4 fibrosis, the proportion of these stages was also low, at 4.4% and 0.7%, respectively.

The evaluation of MASLD in our cohort revealed no significant differences in terms of liver fibrosis or other histological characteristics. However, a difference in hepatic fibrosis could have been anticipated, as the group with a CAP ≥ 275 dB/m exhibited abdominal obesity, one of the most significant factors in the development of advanced fibrosis, cirrhosis, and HCC [14]. In this group, HbA1c levels were significantly higher, which may support the hypothesis that liver steatosis often precedes the development of T2D. Additional studies should be conducted to further investigate the role of impaired glucose metabolism as an early contributing factor to steatotic liver disease..

There is a clear discrepancy between previous reports and our study. One possible explanation could be the duration of obesity. The longer individuals have been living with obesity, the higher their risk of developing comorbidities such as MASLD. Unfortunately, no data were available to assess this factor. Secondly, the proportion of patients with diabetes may also influence the results. In our study, only 16.3% of patients had diabetes at baseline, whereas the studies mentioned earlier reported that up to 44% of their patients had diabetes. Finally, another possible explanation could be the effectiveness of the Dutch healthcare system, which offers free procedures and maintains short waiting times. In contrast, other countries may depend on private healthcare with high costs or have overburdened public systems, leading to delays in patient care and potentially contributing to the progression of MASLD [39].

The use of NITs is helpful for detecting at-risk patients, but our study indicates that there might be an overestimation of the obtained measurements or calculation. The FIB-4 in our study performed significantly worse than in previous validation studies. The AUROC of 0.645 for detecting F ≥ 2 is notably lower than the AUROC of 0.81 reported by Siddiqui et al [40]. In contrast to that study, our patients had a much higher BMI and lower transaminases. Therefore the two cohorts are not comparable and it may be the case that FIB-4 is not a valid test in subjects as included in the present study. TE misclassified one-third of our histology-based MASLD cases. To our knowledge, no validation study has yet demonstrated the accuracy of the capped MAF-5, which was the best performer in our study detecting fibrosis stage ≥2, with AUROCs of 0.809. Only the AUROCs for the APRI and NFS scores were comparable to those reported by Raverdy et al., at 0.75 and 0.68, respectively.

Therefore, in a Dutch bariatric population with low prevalence of advanced fibrosis or cirrhosis, applying these tests to the entire group may be unnecessary. The evaluation of the liver should target individuals who are at high risk for advanced fibrosis or liver cirrhosis and the choice of test should be carefully considered. Future studies will aim to define the profile of these high-risk individuals to ensure accurate detection and diagnosis.

A significant limitation of this study is the small cohort size, with only 77 liver biopsies performed among 220 individuals. The rationale for this approach was the need to follow guidelines to perform liver biopsy based on current diagnostic algorithms [20]. In comparison, Raverdy et al. analyzed 2,346 liver biopsies, providing a much larger dataset. This small sample size resulted in limited diversity across fibrosis stages, with no diagnoses of F3 or F4 and only a few F2 cases. Consequently, questions may arise regarding the accurate assessment of the diagnostic value of NITs. In the current study, the FibroScan assessment suggests an overestimation of the risk of hepatic fibrosis.

## Conclusion

In the current Dutch cohort, the prevalence of MASLD and MASH in patients prior to bariatric surgery was lower than expected. Histological evaluations revealed that the majority had no liver fibrosis, and none were diagnosed with F3 fibrosis or liver cirrhosis. These results suggest that the current population may exhibit characteristics that differ from those reported in previous epidemiological studies. Furthermore, NITs may have limitations in this population. Among them, the capped MAF-5, which demonstrated the best performance, appears to be the preferred test for ruling out significant fibrosis in patients with severe obesity but a relatively low prevalence of MASLD and MASH. However, traditionally reliable tests such as LSM with TE and FIB-4 index performed worse than expected.
